# Syndrome d’hypoperfusion distale ischémique compliquant une fistule artério-veineuse huméro-céphalique

**DOI:** 10.11604/pamj.2017.26.46.10709

**Published:** 2017-01-31

**Authors:** Youssef Alaoui Lamrani, Chakib Maaroufi

**Affiliations:** 1 Service de Radiologie, Faculté de Médecine et de Pharmacie, Université Sidi Mohamed Ben Abdellah Fès, CHU Hassan II, Fès, Maroc; 2 Centre d’Hémodialyse, Hôpital Al Ghassani, Fès, maroc

**Keywords:** Fistule artério-veineuse, hyperdébit, hypoperfusion distale, Arteriovenous fistula, hyperdébit, distal hypoperfusion

## Image en médecine

Madame R.G. âgée de 53 ans, hémodialysée pour néphropathie indéterminée a présenté 9 ans après la confection d'une fistule huméro-céphalique (FHC) gauche, l'apparition d'une douleur chronique de la main homolatérale, associée à des ulcérations nécrotiques des doigts. Un écho-doppler de la FHC a montré un hyper-débit à 1450ml/min, associé à une inversion du flux de l'artère humérale en aval de la fistule. Devant les signes montrant un hémo-détournement vers le shunt artério-veineux, le diagnostic d'un syndrome de vol vasculaire a été porté. La fistule a été ligaturée et un autre shunt artério-veineux a été réalisé sur le membre controlatéral. L'hypo-perfusion ischémique distale ou syndrome de vol peut être le résultat d'une combinaison de lésions sténotiques, d'une artériopathie distale, et de l'inversion de flux en aval du shunt. Non pris en charge, ce syndrome peut évoluer vers la nécrose digitale, voire la nécrose de toute la main. Ses principaux facteurs de risque sont les fistules mises au-dessus de la trifurcation brachiale, le sexe féminin et le diabète. La paresthésie, la douleur au repos, la froideur au cours de la dialyse, et l'atrophie musculaire, représentent des indications formelles pour rétablir le flux vers la main souffrante, et éviter la gangrène digitale. Plusieurs techniques peuvent être proposées pour réduire l'hyperdébit, entre autres, la technique de DRIL correspondant à réaliser une revascularisation distale par pontage veineux et ligature de l'artère en aval de la fistule, une autre technique correspond à la plicature du segment veineux en aval de la fistule pour réduire son débit.

**Figure 1:**
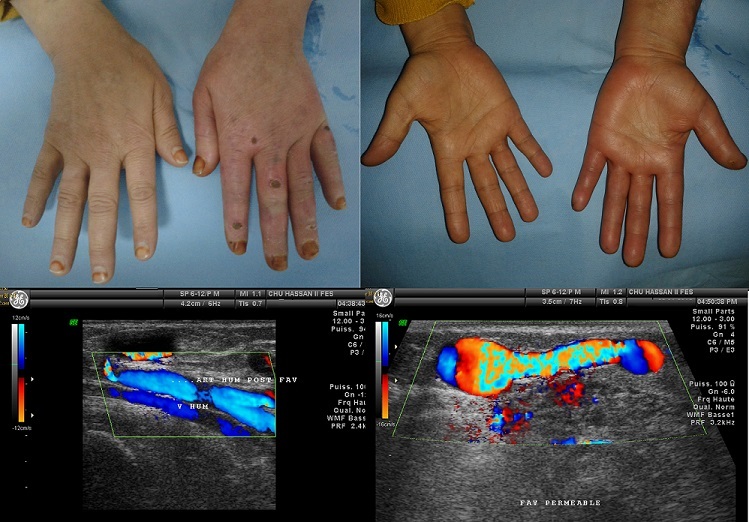
Aspect clinique de la main gauche présentant une coloration un peu bleutée avec des ulcérations digitales nécrotiques; le doppler couleur objective l’inversion de flux au niveau de l’artère humérale en aval de la fistule

